# *Plasmodium falciparum* genomic surveillance reveals spatial and temporal trends, association of genetic and physical distance, and household clustering

**DOI:** 10.1038/s41598-021-04572-2

**Published:** 2022-01-18

**Authors:** Mouhamad Sy, Awa B. Deme, Joshua L. Warren, Angela Early, Stephen Schaffner, Rachel F. Daniels, Baba Dieye, Ibrahima Mbaye Ndiaye, Younous Diedhiou, Amadou Moctar Mbaye, Sarah K. Volkman, Daniel L. Hartl, Dyann F. Wirth, Daouda Ndiaye, Amy K. Bei

**Affiliations:** 1 Laboratory of Parasitology and Mycology, Cheikh Anta Diop University, Aristide le Dantec Hospital, Dakar, Senegal; 2 Department of Biostatistics, Yale School of Public Health, New Haven, CT USA; 3 Department of Immunology and Infectious Diseases, Harvard T. H. Chan School of Public Health, Boston, MA USA; 4 The Broad Institute of MIT and Harvard, Cambridge, MA USA; 5 College of Natural, Behavioral and Health Sciences, Simmons University, Boston, MA USA; 6 Department of Organismic and Evolutionary Biology, Harvard University, Cambridge, MA USA; 7 Department of Epidemiology of Microbial Diseases, Yale School of Public Health, New Haven, CT USA

**Keywords:** Evolutionary genetics, Parasite evolution, Parasite genetics

## Abstract

Molecular epidemiology using genomic data can help identify relationships between malaria parasite population structure, malaria transmission intensity, and ultimately help generate actionable data to assess the effectiveness of malaria control strategies. Genomic data, coupled with geographic information systems data, can further identify clusters or hotspots of malaria transmission, parasite genetic and spatial connectivity, and parasite movement by human or mosquito mobility over time and space. In this study, we performed longitudinal genomic surveillance in a cohort of 70 participants over four years from different neighborhoods and households in Thiès, Senegal—a region of exceptionally low malaria transmission (entomological inoculation rate less than 1). Genetic identity (identity by state, IBS) was established using a 24-single nucleotide polymorphism molecular barcode, identity by descent was calculated from whole genome sequence data, and a hierarchical Bayesian regression model was used to establish genetic and spatial relationships. Our results show clustering of genetically similar parasites within households and a decline in genetic similarity of parasites with increasing distance. One household showed extremely high diversity and warrants further investigation as to the source of these diverse genetic types. This study illustrates the utility of genomic data with traditional epidemiological approaches for surveillance and detection of trends and patterns in malaria transmission not only by neighborhood but also by household. This approach can be implemented regionally and countrywide to strengthen and support malaria control and elimination efforts.

## Introduction

As malaria transmission declines, and as population level immunity wanes, spatiotemporal variation in malaria incidence is likely to become more pronounced^1–4^. This heterogeneity is evidenced by hotspots of malaria transmission which can sustain and amplify the malaria transmission chain^5,6^. Targeting and eliminating malaria hotspots can play an important role in malaria elimination^3,6^. Different studies using conventional malaria epidemiologic tools such as malaria incidence and prevalence rates, malaria morbidity and mortality rates, and entomological inoculation rates (EIR) have identified hotspots of malaria transmission; however, some of these metrics are better suited for more highly endemic regions^7–9^ and may become less useful as malaria transmission declines. As some regions strive for malaria elimination, along with the decline of the disease incidence some of these conventional malaria metrics may become less informative^10^.

Genomic data from sensitive molecular tools are capable of detecting low level parasitemia and of providing additional information on parasite genetic population structure to measure the dynamic changes in malaria transmission^10,11^. Genomic epidemiology has been used to detect associations between malaria parasite genetic diversity, dynamic changes in transmission intensity, and malaria programmatic impact^12–15^. We have previously validated a 24-SNP molecular barcode for monitoring changes in transmission intensity as well as for tracking specific parasite types in the population^16^. More specifically, genomic tools can reveal whether specific genotypes dominate hotspots from focal local transmission of individual strains, or whether the malaria transmission landscape is characterized by increased genetic diversity with significant potential for outcrossing resulting from sustained transmission or importation of multiple genotypes^10,17^. Genomic data can be coupled with mapping data, such as geographic information system (GIS), for visualizing spatial epidemiology. These combined data types can be informative for evaluating control measures. For example, GIS and epidemiologic data were used for mapping clusters of malaria transmission in Gambia, Mali and Senegal and used to guide optimal malaria control intervention^18^. GIS information can also give important information at the household level for studies in micro-epidemiology and ecology^19^. This combined approach was used in Papua Indonesia by coupling genetic information of *Plasmodium falciparum* and *P. vivax* from microsatellite data and household level GIS information to study malaria micro-epidemiology^20^. In Senegal, few studies have used genomic data to understand *Plasmodium* parasite genetic diversity and spatiotemporal dynamics. Here, we seek to bridge this gap by using genomic epidemiology and ecology at the city, neighborhood, and household levels.

The aim of this present study was to apply the 24-SNP molecular barcode in a longitudinal cohort enrolled between 2014 and 2017 and followed for 2 years after enrollment to understand *P. falciparum* parasite population structure in Thiès, Senegal. We determined the spatio-temporal parasite haplotype distribution, and the association between physical distance and parasite genetic distance and the interconnectivity between parasites using a recently developed hierachical Bayesian regression model^21^. These analyses helped generate hypotheses on possible reasons for transmission hotspots. The overall goal of this study is to help inform malaria control by integrating genomic data into decision making.

## Results

### Patient demographics and characteristics of the cohort

A total of 70 participants were enrolled following informed consent spanning 4 years (2014 = 2, 2015 = 32, 2016 = 23 and 2017 = 13) and followed for two years post-enrollment. Patients were recruited through passive case detection upon presenting at the Service de Lutte Anti Parasitaire (SLAP) clinic with malaria-like symptoms and testing positive by malaria rapid diagnostic test (Pfhrp2 antigen RDT) and microscopy for *P. falciparum* monogenomic infection. Patients were residents of 6 different neighborhoods in Thiès (Cité Senghor, Diakhao, Escale, Nguinth, Thialy and Takhikao) and 10 different houses (BD, BS, DL, DMS, GB, MS, OB, OD, SD and SN). The majority of the participants were from Diakhao 51/70 (72.8%), and the majority of shared household participants were in household MS; 37/70 (52.8%) (Table 1). While enrollment was open to all genders and ages, in this study all participants were male aged from 5 to 16 years. When evaluating the gender bias after enrollment, all were living in “daaras” - the equivalent of religious boarding schools. Only 11 of 65 (16.9%) participants reported using a bednet or other forms of mosquito prevention. The mean age was 10.91 years, and 25% (18/70) of the participants were under 10 years. The mean parasitemias were 0.77%, with a minimum of 0.03% and a maximum of 4.89% (Table 1).

**Table 1:** Demographic characteristics of the study population.

Characteristic	Number (%)
**Sex, number (%)**	
Male	70 (100)
Female	0 (0)
Ratio (M/F)	–
**Age, years**	
Mean	10.91
Range	(5–16)
**Parasitemia, (%)**	
Mean	0.77%
Range	(0.03–4.89%)
**Neighborhood, no. (%)**	
Cite Senghor	1 (1%)
Diakhao	47 (67%)
Escale	1 (1%)
Takhikao	5 (7%)
Thialy	5 (7%)
Nguinth	11 (15%)

Patient demographics for the n = 70 participants.

Sex, Age (in years), Parasitemia (calculated from thin smears), and breakdown of participants by neighborhood are shown.

### Limited genetic diversity and high frequency of monogenomic infection

**Figure 1:**
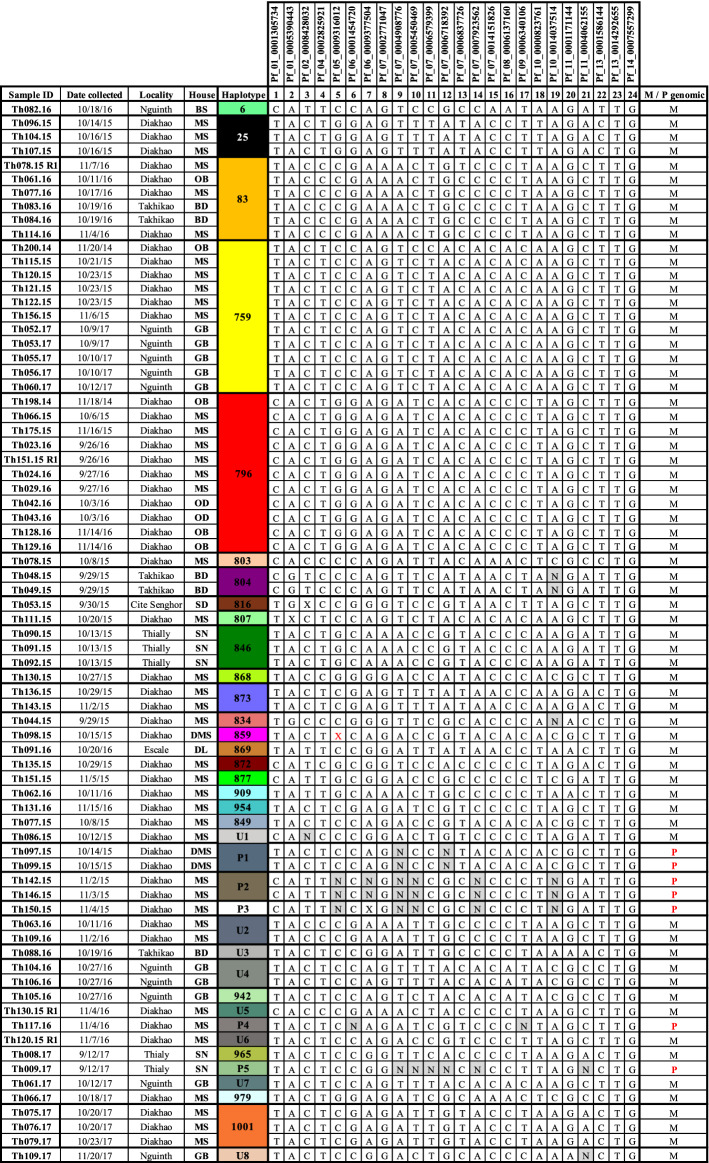
Barcodes of *Plasmodium falciparum* isolates identified in the study cohort. Barcoded parasites from the n = 70 participants in the longitudinal cohort followed for 24 months. Patient ID (human) is shown along with date of sampling, Age, Neighborhood, Household (arbitrary alphabetical code), and barcode haplotype. SNP positions are indicated for the 24-SNP barcode. “N” indicates a position with two peaks—corresponding to both biallelic SNPs (mixed genotype), and an “X” indicates a reproducibly negative call. M/P genomic indicates monogenomic (M) or polygenomic (P) infections.

**Figure 2:**
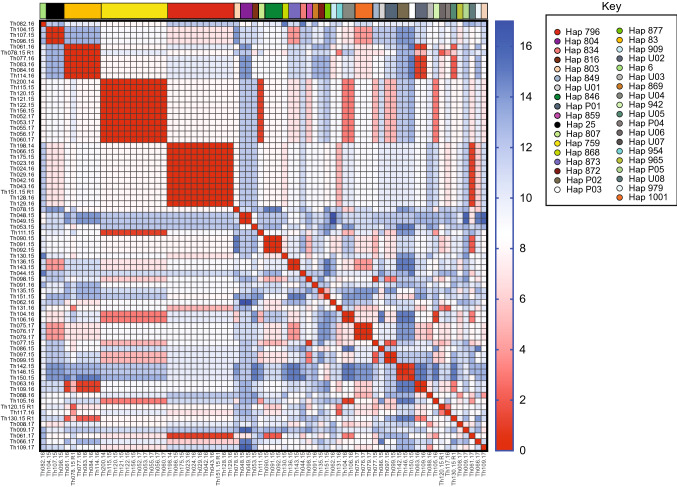
Heatmap illustrating parasite genetic barcode similarity. Sample IDs are listed on the vertical axis and barcodes are clustered by genetic similarity. Barcode haplotypes are color coded at the top of the heatmap and in the Key. Heatmap scale (right) shows the number of barcode SNP differences and range from identical (fewest differences; red) to greatest differences (blue). The maximal number of differences possible is 24.

Among the 70 participants, there was a total of 74 distinct infections. All participants were parasitemic at day 0, and 4 participants were infected with a subsequent re-infection over the course of 2 years of follow-up (8 visits, 4 each year). The participants’ infections were distributed across time as follows: 2014 (2 infections), 2015 (32 infections), 2016 (23 infections, 4 reinfections: 27 total), 2017 (13 infections). Of the 74 infections genotyped, monogenomic infections were predominant 90.5% (67/74) and polygenomic infections were rare, 9.5% (7/74). This finding is predicted for the region^14^. The majority of polygenomic infections were observed in 2015 (5/7). More than 64.8% (48/74) of parasite genetic types were shared within participants of the cohort overall, and when shared by year, 2014 = 0% (0/2), 2015 = 66% (21/32), 2016 = 67% (18/27), and 2017 = 62% (8/13).In comparison with cross-sectional barcode data from Thies from clinical cases identified in 2015, 2016 and 2017, the proportion of shared genetic types between isolates were 48% (80/165),50.4% (62/123) and 35% (35/100, respectively (^14^, and unpublished data). The prevalence of shared genetic types in the cohort was just significantly different from the overall percentage in the population (p =.03) for 2015 and 2017, by Z-test for two population proportions; 2016 was not significant (p=0.06). Of the 74 infections, 32 distinct monogenomic distinct genotypes (haplotypes) were identified. Twenty-four haplotypes previously described in Thiès were detected in 57 isolates (57/74); eight unique genotypes were observed in this cohort and detected in ten participants (10/70). Among the 24 haplotypes we found 8 clusters (shared parasite haplotypes between at least two participants); these were haplotypes 25, 83, 759, 796, 804, 846, 873, and 1001. Among the 10 unique parasites, 2 clusters (U2 and U4) in a total of 4 infections were found (Fig. 1). A heatmap reveals parasite clustering by barcode similarity (Fig. 2), and has also been displayed using hierarchical clustering using Ward’s method (Fig. S1). In this population, average expected heterozygosity ($H_{e}$) was described as a measure of genetic diversity. Using the 24-SNP barcode data, nucleotide diversity (SNP$\pi$) was measured to be 0.274 and $H_{e}$ was found to be 0.371 (95% confidence interval: (0.341, 0.401), indicating an overall low level of genetic diversity. Further, the genotypic richness index *R* was used to describe the proportion of unique genotypes present in the population^22^, and was found to be 0.425, again, indicating low population level genetic diversity among circulating strains.

### Genomic haplotypes are shared within years and persist across malaria transmission seasons

**Figure 3:**
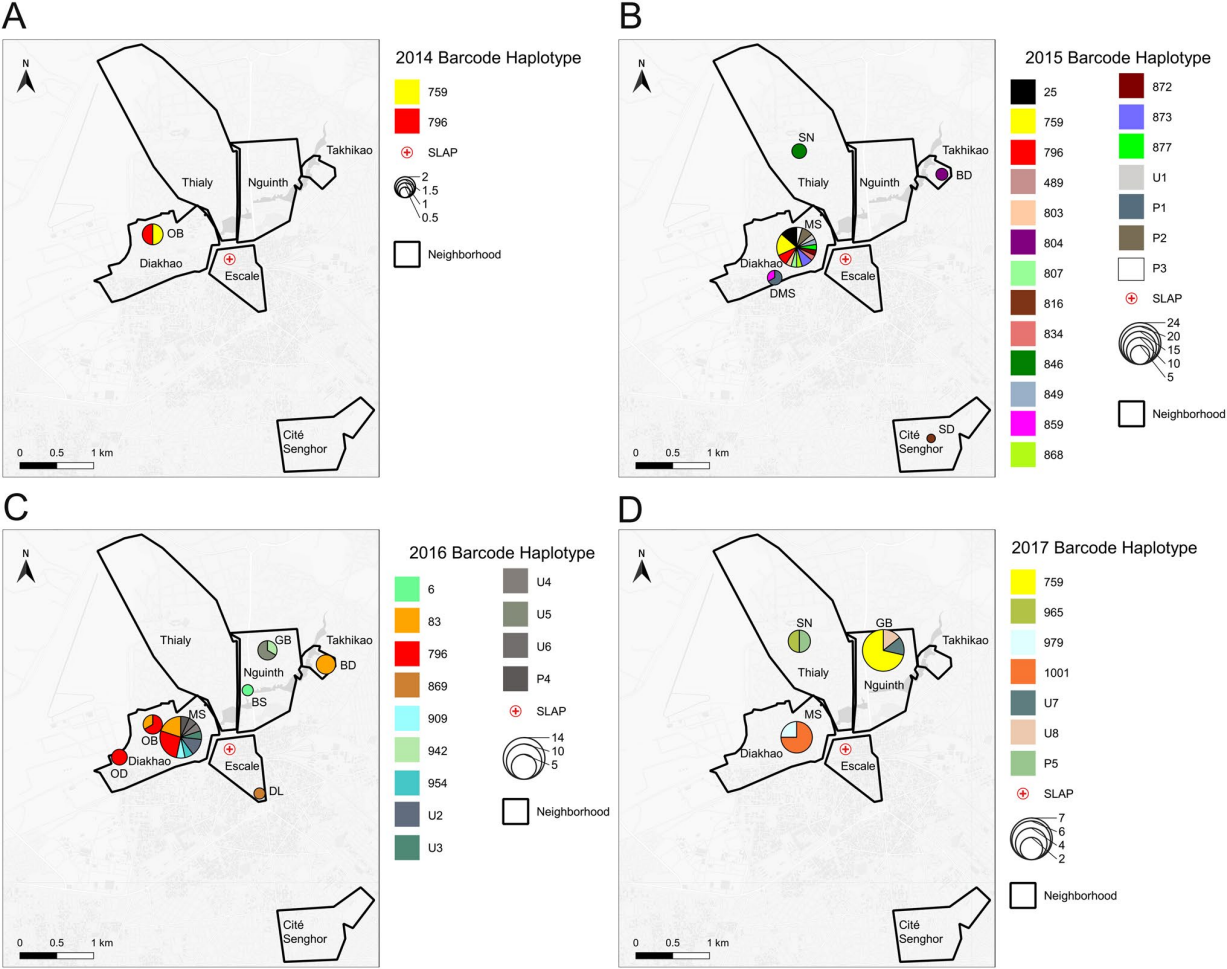
Hotspots of malaria transmission by neighborhood and household. Map of sample locations by household (BD, BS, DL, DMS, GB, MS, OB, OD, SD and SN), and neighborhood (Cité Senghor, Diakhao, Escale, Nguinth, Thialy and Takhikao) from 2014 to 2017. Legend indicates and the parasite barcode haplotype ID and is color-coded by haplotype. The size of the circle is proportional to the number of samples in the haplotype, as indicated in the scale. Solid lines indicate delineation of the neighborhoods.

Genetic types were shared within a transmission season but also across transmission seasons (lineage persistence). The dominant haplotypes that persisted for multiple seasons were haplotypes 759 and 796. Haplotype 759 (11/70) was detected in 2015 in Diakhao in the MS household (5/32) and in 2017 in Nguinth in the GB household (5/13). Haplotype 796 (11/70) was observed in 2014 (1/2), 2015 (both in MS) and 2016 (8/23) in the neighborhood of Diakhao in three different households (MS, OB and OD).Those two haplotypes are also the predominant haplotype persistent over year (2015, 2016, and 2017) in the Thies barcode data base (data not published), with haplotype 759 detected all 3 years. Haplotype 83 (6/70) was found in 2016 in two different neighborhoods (Diakhao and Takhikao) in three different households (MS, OB and BD) (Fig. 3).

### Genomic signatures among initial and re-infection

**Figure 4:**
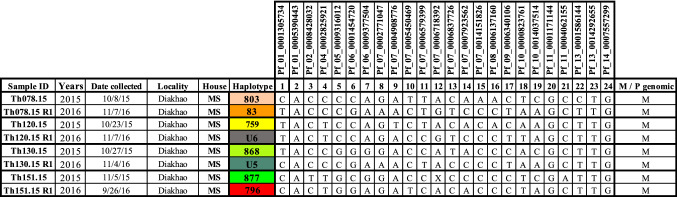
Barcodes of initial and re-infected participant samples. Patient ID (human) is shown along with date of sampling, Age, Neighborhood, Household (arbitrary alphabetical code), and barcode haplotype. SNP positions are indicated for the 24-SNP barcode. “N” indicates a position with two peaks—corresponding to both biallelic SNPs (mixed genotype), and an “X” indicates a reproducibly negative call. M/P genomic indicates monogenomic (M) or polygenomic (P) infections.

During the patient follow-up, 4 participants recruited in 2015 (Th078.15, Th120.15, Th130.15 and Th151.15) were re-infected in the following year during the peak of the malaria transmission period. Unscheduled visits in which study subjects had symptomatic infections are designated as “re-infections” or R1 (Th078.15.R1, Th120.15.R1, Th130.15.R1 and Th151.15.R1). Re-infected participants were all from the same household (MS). Parasite genotypes from the first infection were genetically distinct from the re-infected parasites (Fig. 4), and three of them (U5, U6 and U7) represented genotypes which had not been previously described, either in Thiès or in multiple regions of Senegal from 2006 to present^14,16,23^.

### Effect of spatial distance on parasite genomic similarity, measured by IBS

We calculated the genetic similarity between each unique pair of patients as well as the geographic distance between their respective households to determine whether increasing physical distance between households is associated with greater genetic difference. When analyzed by year (to normalize for the circulating genetic variants present within a season), we observed a significant positive association between physical and genetic distance in 2015 and 2016 (Table 2) even after adjusting for correlation between the paired responses; as physical distance increased between two households, so did the genetic distance of the patients in those households (increase of 31% (95% credible interval, (11, 58)) per km increase in separation in 2015 and 16% (95% credible interval, ((2, 31)) in 2016). When data were analyzed within a year (to account for differences in the genetic types most prevalent between years), we observed a similar trend (increase of 4% (95% credible interval, (0,8)) per km increase in separation). Further, we observed that in three of five households (the exceptions being households MS and OB), living in the same household was more likely to result in participants being infected with similar genomes (i.e., exponentiated regression parameter estimates smaller than one), although these findings were not generally statistically significant, likely because of the small number of individual pairs within some of the households (Table 2). In 2015, we were unable to estimate household-specific associations because of low sample sizes but did not see significant clustering in household risk overall.

**Table 2:** Relationship between physical distance and genetic distance.

	Estimate	95% Credible interval
**Effect (2015)**		
Distance (km)	1.31	(1.11, 1.58)
Same house	0.92	(0.76, 1.11)
**Effect (2016)**		
Distance (km)	1.16	(1.02, 1.31)
House 1: BD	0.12	(0.02, 0.49)
House 2: GB	0.56	(0.25, 1.27)
House 3: MS	1.29	(0.96, 1.74)
House 4: OB	1.32	(0.62, 2.77)
House 5: OD	0.35	(0.00, $\infty$)
**Effect (2017)**		
Distance (km)	0.92	(0.36, 2.22)
House 1: GB	0.34	(0.03, 3.17)
House 2: MS	0.41	(0.07, 2.30)
House 3: SN	0.63	(0.14, 2.70)

The relationship between physical distance (measured in kilometers (km) between two GPS coordinates) and genetic distance (measured as identity by state, the number of barcode differences in the 24-SNP molecular barcode) are shown overall, as well as by household.

Estimates (posterior medians) are shown for the exponentiated regression parameters along with 95% quantile-based equal tailed credible intervals.

### Effect of temporal distance on parasite genomic similarity, measured by IBD

**Figure 5:**
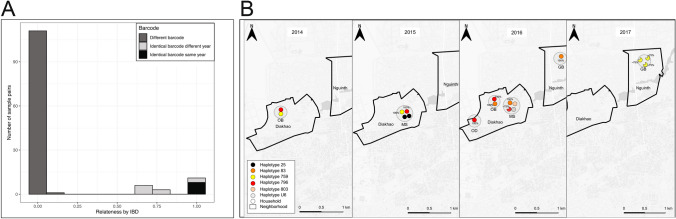
Identity by Descent reveals subtle differences among identical barcode samples separated in time. (**A**) Relationship between identical barcode parasites (measured by IBS) and IBD with time. Percent of whole genome relatedness by IBD, color-coded by barcode identity (IBS), identical or different. (**B**) Relationships between 17 specific identical barcode haplotypes by household, year, and IBD, determined from whole genome sequencing. Years are shown on the X axis. Colored circles represent individual parasites from haplotypes that are color coded according to barcode haplotype. Pie charts and percentages shown represent percent IBD relative to the pairwise comparison to the initial isolate, as defined temporally.

We next sought to compare whether in this specific population of a lowly endemic region of Senegal, if IBD among parasites that are identical by barcode was highly correlated in space (household) and time (transmission seasons) (Fig. 5). For samples with different barcodes, they also showed minimal (or no) identity by IBD. Samples with identical barcodes fell into two categories, those that had complete (100% IBD) and those with lesser IBD. The samples with lesser IBD (ranging from 70 to 75%) were exclusively found separated by year, whereas samples from the same year and different years were found to have 100% IBD. (Fig. 5) This result indicates that identical barcodes within a transmission season are more likely to be more similar and share a greater percentage of their genome than those that are separated in time.

## Discussion

As malaria control progresses towards elimination, genomic data has proven to be essential in assessing control and surveillance^24^. In this study we combine parasite genetic diversity indices, individual global position system (GPS) information at the neighborhood and household level, and a hierarchical Bayesian regression model to understand parasite diversity and connectivity over time and space in Thiès, Senegal. The main findings of this study were household clustering of genetic types, association with genetic distance and physical distance, as well as parasite sharing between participants from either the same household or different households which were geographically proximal. This study provides additional data from a well characterized low transmission setting at very focal spatial scales and with precise mapping of malaria in households and neighborhoods, specifically in *daaras*. This fine spatial and temporal scale is not always possible with large cross-sectional datasets; and interestingly, in past analyses from cross-sectional studies in Thiès, this spatial clustering of identical genotypes was not observed^25^.

Overall, the low level of *Plasmodium* parasite genetic diversity and the high frequency of monogenomic infection observed over years are generalizable and consistent with previous observations from cross-sectional sampling over time in Thiès^14,25^ and Dielmo and Ndiop in Senegal^16^. Average expected heterozygosity ($H_{e}$), a common measure of parasite genetic diversity, represents the probability of being infected by two parasites with different alleles at a given locus. The value of $H_{e}$ in this population was found to be 0.371 (95% CI (0.341, 0.401)). Coupled with the genotypic richness (0.425), relatively much lower than has been described in Malawi^26^ and in regions of declining transmission on the Thai-Burma border^22^, these measures emphasize the low genetic diversity in this population. In these localities 24-SNP molecular barcoding revels a predominance of monogenic infection and a significant percentage of shared genomic haplotypes in the population. These observations have been hypothesized to be the result of a significant reduction in malaria transmission due to the efficiency of malaria interventions post-2008.

Consistent with previous studies in Thiès, we also observed the existence of haplotypes persisting over several years^14,25^. Incorporating focal GIS data permitted us to monitor the genotype frequencies in different households nested within neighborhoods within the same year, and across seasons. For example, haplotype 796 was observed in the same neighborhood (Diakhao) in 3 years (2014, 2015, and 2017) in three different households (OB, OD, and MS). Similarly, haplotype 759 was observed in two different neighborhoods, in 3 successive years (2014-2017) and in three different households (OB, OD, and MS). When observing identical genotypes in households, there are two possibilities: 1) continued local transmission of a single parasite genotype that maintains genomic identity through selfing, or 2) the same infected mosquito biting multiple individuals within the household or neighborhood. The spatial and temporal nature of the infections can help distinguish which hypothesis is more likely; but additionally, this is an area where IBD has added value. Clonal propagation of identical parasites in households over short temporal scales (days) would favor infection of multiple individuals by the same infected mosquito, yielding identical parasites by both IBS and IBD. Continued focal transmission of identical parasites in households observed over longer temporal scales (weeks to months to years) would favor local household transmission of inbred parasite lineages, and here we may expect more heterogeneity in IBD even in parasites that are IBS due to limited outcrossing in the mosquito. We observe both scenarios in our study. An increase in genetic diversity and limited clonal propagation would imply imported parasites followed by outcrossing or co-transmission, with outcrossing resulting in genetically diverse monogenomic infections and co-transmission resulting in an increase in polygenomic infections. This study has also demonstrated that household transmission of the same genotype is frequent in Thiès, and that cross-neighborhood and cross-year transmission of the same genotype is also common, again implying a relative lack of outcrossing in the overall population^27^.

As the overall degree of genetic variation can vary depending on the season of transmission, the intensity of transmission, and also the degree to which new strains are introduced into the population^28^, genomic surveillance by longitudinal sampling can provide valuable insights into changing malaria ecology and transmission dynamics. Interestingly, but perhaps not unexpectedly, the genetic similarity of parasites identical by barcode (IBS) breaks down a bit when assessing genetic similarity at the whole genome level through IBD. IBD analysis shows that for haplotypes persisting for multiple years, identical parasites which are clonal by IBS most often are identical at the whole genome level, but can share from 70 to 100% of their whole genome, with those more distantly related in time sharing less overall genomic identity. We observed this notably in two instances, one of a haplotype spanning a two year gap and identified in a more distant household (Haplotype 759; 2015—Household MS and 2017—Household GB), and the second in a haplotype spanning multiple years (2014–2015–2016), and multiple households, but yet IBD was only decreased in the isolate from the highly diverse household MS. This finding implies that some of these barcode clonal parasites experienced some degree of out-crossing, and this was more likely over time and in households with a large and diverse pool of parasites serving as a potential recombination reservoir.

We observed household clustering and genetic differences between parasites to increase with distance between individuals. During this study, a particular household (MS) served as an example of a malaria hotspot of transmission at the household level, both in number of cases as well as genetic diversity of the parasites. Having such different parasites in the same household could be the result of importation of diverse genotypes due to human or mosquito mobility^23,29–32^, followed by genetic recombination (outcrossing) within the *Anopheles* mosquito^33^, and the subsequent transmission of new genetic combinations^17^ resulting in a hotspot of local intense transmission^17^. The predominance of polyclonal infections in this household would also favor this hypothesis. A similar study of malaria incidence and prevalence has demonstrated the existence of malaria transmission hotspots at the village level in Senegal^7,8^. In such villages, human density, human behavior, infrequent malaria bed-net use, substandard housing construction, and a favorable ecological environment for mosquito proliferation (presence of mosquito breeding sites) have all been identified as risk factors for a household to be in a hotspot^34^. The added value of our approach is being able to identify hotspots of transmission, but also to determine the genotypic nature of these hotspots - adding further to implications for control measures. If hotspots are populated by similar genotypes, it is more likely that local transmission of selfing strains is occurring. If multiple diverse monogenomic genotypes are present, the hotspot could serve as a hub of human or mosquito imported infections. If polyclonal infections increase, it implies a combination of both: importation following by increased local transmission. Identification of the transmission clusters at the household level will play an important role for interrupting malaria transmission chains^5,35^. Identifying neighborhoods or households with high malaria transmission can assist malaria control programs with focal interventions to reinforce malaria prevention and control.

Because *P. falciparum* is a sexually recombining organism, precise mapping of phylogeny and transmission chains is not possible; however, the 24-SNP barcode has been shown to be a proxy for whole-genome that allows resolution especially of highly similar parasite types^14^. While the 24-SNP barcode does not provide as complete information about genetic relatedness (identity by descent) as whole-genome sequencing or large SNP arrays^36^, it has been estimated that the 24-SNP barcode can confidently detect parasites that share greater than 70% genome similarity (identity by state)^14^. While the pairwise genetic distance in the 24-SNP barcode is not linearly associated with whole-genome genetic distance, our finding of significant associations with physical distance is even more noteworthy. Our statistical model demonstrated that genetic variation between parasite pairs increases with physical distance. Here we used the number of SNP differences between paired individuals as genetic distance, or identity by state. Studies in The Gambia and Kenya have demonstrated that variation between parasite genotypes increases with geographical distance^37,38^. Such findings will help in understanding how the parasite population is structured in Thiès and the connectivity between parasites, despite some studies in Thiès having suggested a mixed parasite population with no hidden population structure^27^. In this study, sampling biases (number of limited samples) may not reflect the overall parasite population that is captured by passive case detection, and notably, we found no asymptomatic infections in any of the follow-up time points in the cohort.

All of the enrolled participants in our study live in “*daara*”s, religious boarding schools where “*talibe*” (resident student followers) live together in large numbers. One particular *daara*, arbitrarily termed “MS” had a very large proportion of cases, a diverse haplotypes and all re-infected participant were from that household. As a specific population, little is known regarding the malaria burden in this specific community of children, although it has been proposed that this population is considered more vulnerable and may have higher risks of parasitic infectious diseases, due to living conditions^39^ The high malaria burden in this community may be explained by the household size, close living and sleeping quarters, socio-demographic factors, and less consistent compliance with long-lasting insecticidal net (LLIN) use^40^. The Senegal national malaria program control (NMCP) has recently established a malaria case management at the level of *daraas* (PECADARA) to screen and treat students living in these boarding residences with the ultimate goal of preventing morbidity, mortality, and transmission in this demographic. Our findings of malaria burden, evidence of multiple infections of the same parasite in the same household, as well as some households with highly diverse infections, implying a “melting pot” for imported types and recombination^23^, all support the notion that extended malaria surveillance specifically in the *daaras* could be an important strategy to prevent continued malaria transmission chains in the community. *Daaras* also represent an attractive opportunity for intervention for NMCPs as there is the opportunity to systematically reach many children living in the same household.

Our study has some limitations. Our cohort was completely male, although enrollment was open and encouraged for both male and females. The ages of participants were children and adolescents, sampling was limited to the high transmission season, and malaria infections were all symptomatic and detected by passive case detection. As the participants were enrolled in a longitudinal cohort and followed over time, we may have observed selection bias for more solitary individuals as those who intended to travel may have opted not to participate. As previously described, all of the samples from participants in this study came from residents of *daaras*, thus our results would be generalizable to other male residents of *daaras*; however not to the general population. Yet, this study is the first to provide a detailed genetic characterization of the parasite populations in *daaras* in Senegal and will provide valuable information to the Senegalese NMCP which is implementing specific interventions in *daaras* this year. Going forward, studies prospectively designed to specifically investigate malaria transmission dynamics and population genetics in *daaras* should intentionally include and enroll *daaras* with male children and an equal number of *daaras* with female children. Future studies could also apply the same methodologies, but in a population-based cross-sectional sampling approach, in both the high and low transmission periods, and outside the clinical setting, to capture the genetic complexity of both symptomatic and asymptomatic infections throughout the general population.

Additionally, our sample size of 70 participants with 74 infections over 3 years is relatively small. From previous cross-sectional studies in Thiès spanning detectable signals of declining and rebounding transmission intensity^14^, the mean number of samples (monogenomic and polygenomic) across years was approximately 170 and the mean number of monogenomic samples was approximately 125. Based on this data, we estimate that 100 monogenomic samples would be sufficient to detect subtle changes in transmission intensity over time. This sample size is ideal, but as malaria transmission declines in pre-elimination zones, it might prove difficult to achieve, highlighting the need for complimentary measures of transmission intensity such as serological markers of recent compared to historical past malaria exposure.

While the 24-SNP molecular barcode does have limitations in its ability to infer transmission levels and population connectivity, especially on highly local scales; as evidenced by this study and others^14,23,41^, the 24-SNP barcode can be a useful, and importantly field-deployable tool for rapid assessment of *Plasmodium* genomics. It can be useful in distinguishing polygenomic infections from monogenomic infections for measures of complexity of infection (COI)^16^, which increases with transmission intensity, even if it is unable to distinguish the identities of parasite genotypes within these complex infections. However, these simple genetic metrics still have value in the context of real-time genomic surveillance efforts and can provide useful and actionable data on transmission hotspots, probable importation or local transmission, as well as assessment of the impact of specific interventions aimed at decreasing malaria transmission. While whole genome sequencing and identity by descent provide a wealth of high-resolution genomic information to clarify population genetic connection and potentially transmission chains; at the moment, measures such approaches have not been actionable in real time. Taken together, our study provides important information in the micro-epidemiology of parasite population structure in space and time in daaras in Thiès. The study also provides evidence of the feasibility and power of including genomic analyses, with field-deployable methods performed on site, in making public health decisions.

In conclusion, *Plasmodium* spatial-temporal clustering at the household and neighborhood level were observed along with increasing genetic distance between parasites as a function of physical distance. The longitudinal study shows the importance of applying molecular surveillance along with spatial and temporal modeling to detect hotspots of malaria transmission at fine spatial scales.The value of genomic data is especially powerful when traditional epidemiologic measures of transmission are not available or are limited. Taken together, this work emphasizes the added value of combining traditional epidemiology data, including case investigation, household surveys, climate data, and travel history with genomic data and high-resolution temporal and spatial (GPS) data. Combined, they provide powerful insights into local transmission dynamics. These local patterns can have practical implications in providing data to NMCPs on ways to better target local interventions in a way to maximize impact. Identifying the degree to which sustained local transmission or continuous importation of cases from outside a community can influence the specific policy approach adopted, from a focus on specific household or neighborhood malaria prevention efforts to a focus on human mobility as the dominant driver of transmission. Such insights are facilitated by the rapid, real-time acquisition, analysis, and reporting of genetic data to malaria policy makers and represent an attractive model for integrating malaria genomics into decision-making strategy.

## Methods

### Ethics statement

Ethical approval for this study was granted by the National Ethics Committee of the Ministry of Health in Senegal (Protocol SEN 14/49), the Institutional Review Board of the Harvard T.H. Chan School of Public Health (IRB 14-2830), and the Human Investigation Committee of Yale University (Protocol 2000023287). All samples were collected with informed consent and in accordance with all ethical requirements of the National Ethics Committee of Senegal, Institutional Review Board of the Harvard T.H. Chan School of Public Health, and the Harvard, and the Human Investigation Committee of Yale University.

### Inclusion and exclusion criteria

Samples were collected through passive case detection from patients greater than 6 months of age with symptomatic uncomplicated *P. falciparum* malaria confirmed by a peripheral blood smear and rapid diagnostic test (RDT) by the local health officer of the health clinic of Thiès (SLAP). Patients were asked to return to the health post, regardless of health status, at days 1, 2, 3 and after 2 weeks, 4 weeks, 3 months, 6 months, 12 months, 18 months, and 24 months. Information on intention to travel and number of months in residence at the household was collected, both as part of the enrollment questionnaire, and also as an optional reason an eligible participant decided not to participate. Patients were also asked to return for an unscheduled visit if they experienced malaria-like symptoms. At the visit on days 1-3, the patient was monitored for the clearance of parasitemia by finger prick and a microscopy slide and an RDT was evaluated. At scheduled follow-up visits at 2 weeks, 4 weeks (1 month), 3 months, 6 months, 12 months, 18 months, and 24 months, 5 mLs of blood was drawn for plasma and PBMCs. On Day 0 and at unscheduled visits where a patient was confirmed to be positive with *P. falciparum*, blood was also cryopreserved, and parasite DNA was extracted from whole blood with the QIAamp DNA blood mini kit (Qiagen Inc., Valencia, CA, USA).

### Molecular barcoding genotyping

24-SNP molecular barcodes were identified using a previously described assay^41^. Barcode assays were run on the LightCycler 96 Roche system. SNPs were amplified as follows; $2.0\ \mu$L of Lightscanner Master Mix (BioFire Defense), $2.5\ \mu$L of a 1:100 dilution DNA template, and $0.5\ \mu$L of primers and probes. Genomic DNA from cultured *P. falciparum* strains (3D7, Dd2, 7G8, Tm90) was used for assay validation and as genotyping controls for all reaction plates. Molecular barcode assays 10, 11, 13, 21, and 24 were performed optimally under asymmetric forward to reverse primer ratios of 5:1; all other assays required a 1:5 primer asymmetry. Amplification conditions were 95 $^\circ$C denaturation for 2 min, 50 cycles of 94 $^\circ$C for 5 s and 66 $^\circ$C for 30 s, plus a pre-melt cycle of 5 s each at 95 $^\circ$C and 37 $^\circ$C. Two or more N’s among the 24 SNPs assayed was taken to indicate that more than one *P. falciparum* genomes was present (a polygenomic infection).Polygenomic infections was established in the parasite population by examining the number of heterozygous SNPs (N) in each sample assayed. Infection was classify as polygenomic, when the barcode has at least two (2) heterozygous SNPs (N). Samples were classify monogenomic infections if they have at no more than one (1) heterozygous SNPs (N) in the barcode. Ambiguous calls and calls with “X” were repeated 3 times in independent experiments before validation^41^. All barcoding was performed in Senegal.

### Whole genome sequencing

We performed selective whole-genome amplification (SWGA)^42^; on total extracted DNA. Amplified material was sequenced using Illumina technology with paired-end reads after library construction with a NEBNext Ultra II library prep kit. We aligned reads and called variants following best practices established by the Pf3k consortium. In short, we aligned reads to the *P. falciparum* 3D7 reference genome (PlasmoDB v. 28) using BWA-mem, marked duplicate reads with Picard Tools, and called variants using HaplotypeCaller in GATK v.3.5. We performed variant and base score recalibration in GATK using variants from a set of lab-generated crosses. We prioritized 23 isolates for whole genome sequencing representing the following characteristics: 1) Same barcode, same year, same household; 2) Same year, same barcode, different household; 3) Same barcode, different year, same household; and 4) Same barcode, different year, different household to preliminarily investigate IBD across space and time. Of these, we retained 17 samples that had at least 20% of the genome covered at a read depth of 5x or greater for downstream analysis (Th200.14, Th078.15, Th078.15 R1, Th096.15, Th107.15, Th120.15, Th120.15 R1, Th121.15 Th151.15, Th151.15 R1, Th175.15, Th083.16 , Th114.16, Th129.16 , Th052.17, Th056.17, Th060.17). For each sample, we masked from analysis heterozygous calls and calls with fewer than 5 supporting reads. Identity-by-descent (IBD) was estimated between all pairs of these 17 samples using hmmIBD^28^. As model input, we used a set of 15,075 SNPs with population-level allele frequencies calculated from a large collection of Senegal samples (https://www.malariagen.net/projects/pf3k, and Stephen Schaffner, personal communication).

### Population genetics and statistics

Barcode sequences were aligned using Geneious Prime 2021.2.2. Nucleotide diversity (SNP$\pi$) was calculated using DnaSP Version 6.12.03. The average expected heterozygosity ($H_{e}$),also known as Nei’s genetic diversity, was calculated using ARLEQUIN software version 3.5.2.2 with the following formula, as has been previously described^43^:

$$\begin{aligned} H_{e}= & {} \Sigma (h_{j}/L),\\ h_{j}= & {} (1-p^2-q^2) \end{aligned}$$

where $h_{j}$ = heterozygosity per locus, p and q = allele frequencies for biallelic loci, $H_{e}$ = average heterozygosity for several loci, and L = total number of loci. Here, average $H_{e}$ over all 24-loci represents an estimate of the extent of genetic diversity in the population.

We measured the genotypic richness index, *R*, to describe the proportion of unique genotypes present in the samples, using the following equation, as has been previously described^22^:

$$\begin{aligned} R = (G - 1)/(N - 1) \end{aligned}$$

where G is the number of distinct genotypes and N is the sample size.

We measured the genetic difference by IBS using a SNP difference matrix, Hierarchical clustering was performed using JMP pro (version 15.0.0).

To evaluate the fraction of pairs related by year, we generating confidence intervals by sub-sampling, as previously described^14^ using an existing, larger set of barcode data from Senegal. For each year, we performed 1000 independent sampling iterations and have compiled 68% confidence intervals, representing the mean +/− 1 std deviation (1 sigma), and 90% confidence intervals with the fraction of pairs that are related (Table S1).

### GIS analysis and statistical modeling

GPS coordinates of participants’ households (while not revealing individual participant addresses or identifiable locations) and neighborhoods were used to make different maps with QGIS 3 (http://www.qgis.osgeo.org). We used a recently developed hierarchical Bayesian regression model to determine if the genetic similarity between pairs of participants is related to the geographic distance that separates them^21^. The model accounts for correlation in the genetic distances due to the fact that an individual is involved in multiple paired responses as well as spatial correlation between responses, and has been used in previous work aiming to estimate associations between genetic and spatial distance^44^. The number of 24-SNP barcode differences between each unique pair of participants was used to describe their genetic similarity. We used two metrics to describe spatial proximity in the analysis. First, for each unique pair of participants we determined whether the individuals were located in the same house and if so, noted which house it was. Next, we calculated the geographic distance between the house centroids for each pair. In this way, we explore the impact of geographic distance on genetic similarity in two ways; whether people clustered in the same house are more genetically similar and whether individuals in houses that are closer together geographically are more genetically similar.

We then model genetic distance between each pair of participants as a function of the spatial distance between their houses and a clustering indicator for the specific house, where each house has its own specific regression parameter. The model is given as

$$\begin{aligned}&Y_{ij}|r,p_{ij} {\mathop {\sim }\limits ^{{\text {ind}}}}\; {\text {Negative Binomial}}\left( r, p_{ij}\right) ,\\&{\text {logit}}\left( p_{ij}\right) = \beta _0 + \beta _1 d_{ij} + \sum _{k=1}^m \alpha _k 1\left( {\text {House}}_i = {\text {House}}_j = k\right) + \theta _i + \theta _j \end{aligned}$$

where $Y_{ij}$ is the genetic distance between participants *i* and *j*, $r>0$ is the dispersion parameter where small values indicate overdispersion in the data (i.e., variance larger than the mean), $p_{ij} \in \left( 0,1\right)$ defines the mean/variance of the genetic distance distribution with a large value resulting in larger expected genetic distance, $d_{ij}$ is the geographic distance between the house centroids of participants *i* and *j*, *m* is the total number of unique houses in the analyzed dataset, 1(.) is an indicator function that is equal to one if the input statement is true and is equal to zero otherwise, and $\theta _{i}$ are spatially correlated, person-specific random effects that account for the multiple sources of correlation in the data. This model relaxes the assumption that clustering in any house has the same impact on genetic similarity and allows for the possibility that this effect changes across the different houses (i.e., ${\alpha _k}$). The parameter ${\beta _1}$ describes the association between genetic and geographic distance between houses. We fit this model to each individual year of data separately using the SNP function within the GenePair R package (https://github.com/warrenjl/GenePair) and present posterior inference (i.e., posterior medians represent point estimates for parameters and 95% quantile-based equal tailed credible intervals describe uncertainty in the parameters) for the model parameters using 10,000 samples from the joint posterior distribution after removing the first 10,000 draws prior to convergence of the model and thinning the remaining 100,000 by a factor of 10 to reduce posterior autocorrelation. We present inference for the exponentiated regression parameters which represent the ratio of expected genetic distances per specified change in each covariate value. For example, the distance estimate in Table 2 from 2015 of 1.31 suggests that genetic distance is 31% larger between individuals for each km increase in distance between their households. Full details on the statistical model, including prior distributions, are given in^21^.

## Supplementary Information

Supplementary Information.

## Data Availability

Data associated with this manuscript can be found at: https://doi.org/10.5061/dryad.wh70rxwmk.
