# Supplementary material for: Plasmodium falciparum genomic surveillance reveals spatial and temporal trends, association of genetic and physical distance, and household clustering
Source: Sci Rep. 2022 Jan 18;12:938. doi: 10.1038/s41598-021-04572-2 (PMC8766587; doi:10.1038/s41598-021-04572-2)
Supplement: Supplementary file 1 — Supplementary Information. [file 41598_2021_4572_MOESM1_ESM.pdf]

# Supplementary Information for

## *Plasmodium falciparum* genomic surveillance reveals spatial and temporal trends, association of genetic and physical distance, and household clustering

**Mouhamad Sy, Awa B. Deme, Joshua L. Warren, Angela Early, Stephen Schaffner, Rachel F. Daniels, Baba Dieye, Ibrahima Mbaye Ndiaye, Younous Diedhiou, Amadou Moctar Mbaye, Sarah K. Volkman, Daniel L. Hartl, Dyann F. Wirth, Daouda Ndiaye, Amy K. Bei**

**Amy Kristine Bei**
**E-mail: amy.bei@yale.edu**

**This PDF file includes:**

Fig. S1
Table S1

| Year | Point Estimate | 68% CI | 90% CI |
|---|---|---|---|
| 2015 | 0.054 | (0.029, 0.076) | (0.022, 0.099) |
| 2016 | 0.138 | (0.088, 0.187) | (0.064, 0.231) |
| 2017 | 0.197 | (0.095, 0.286) | (0.067, 0.400) |

**Table S1. Fraction of Related Pairs by Year**

Point estimates of the fraction of related pairs by year were calculated by sub-sampling with 1000 iterations per year. We have performed this analysis for the 3 years with sufficiently large sample sizes, namely 2015, 2016, and 2017 and have compiled 68% confidence intervals, (representing the mean +/- 1 standard deviation (1 sigma)), and 90% confidence intervals.

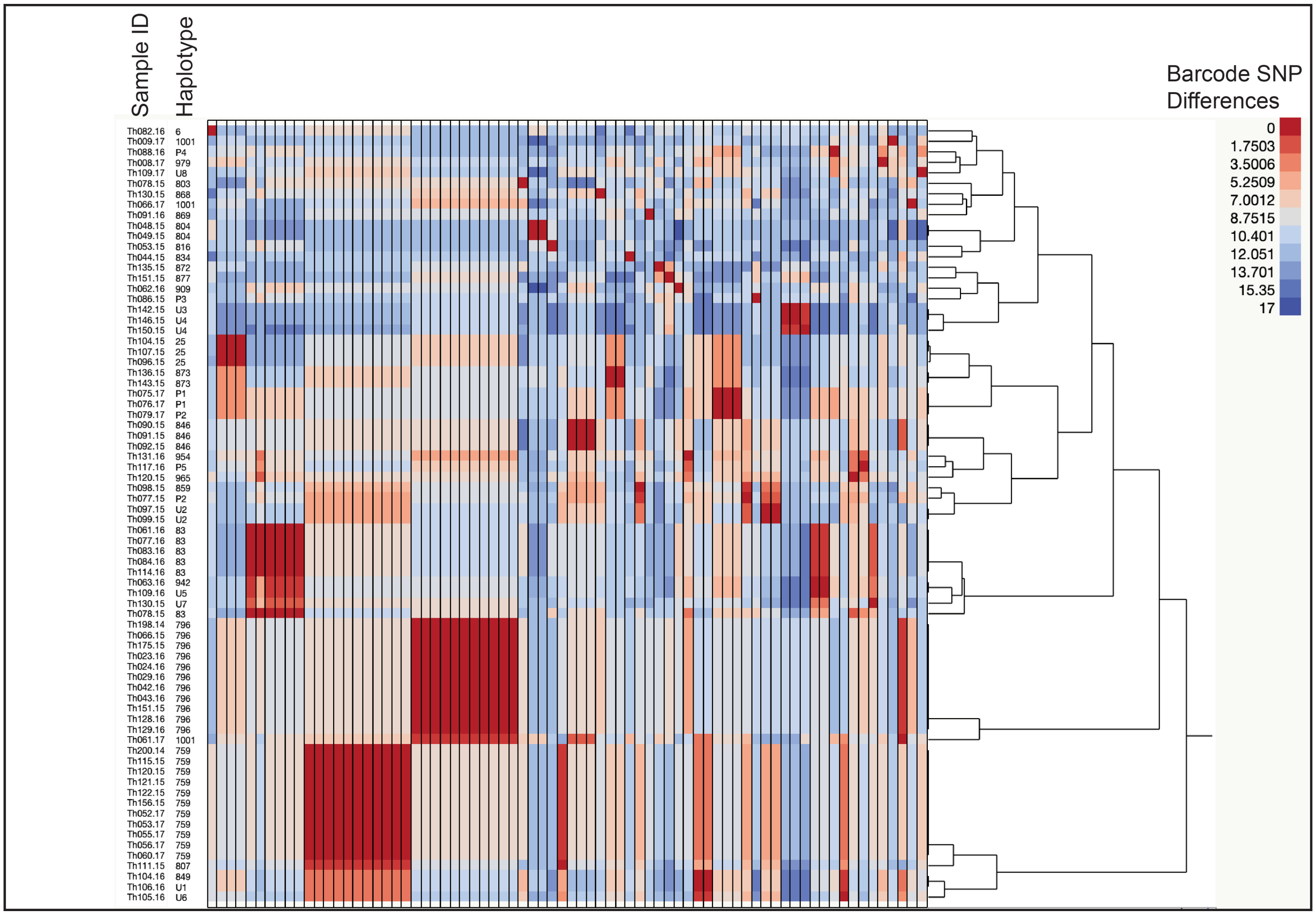


**Fig. S1. Hierarchical clustering of Haplotypes according to IBS difference**

Barcode SNP differences (IBS differences) were used to perform hierarchical clustering using Ward's method to identify clusters of barcode haplotypes by similarity. Hierarchical clustering was performed using JMP pro (version 15.0.0).
